# Therapeutic Potential of Modulating Gene-MicroRNA Crosstalk in Burn Injury

**DOI:** 10.3390/ijms262010060

**Published:** 2025-10-16

**Authors:** Dariya M. Badanina, Anastasia M. Bubnova, Dmitry S. Kozlov, Dmitry P. Krylov, Artem M. Mozherov, Massoud Vosough, Peter S. Timashev, Daria S. Kuznetsova

**Affiliations:** 1 Institute for Regenerative Medicine, Sechenov First Moscow State Medical University (Sechenov University), 8-2 Trubetskaya Str., 119991 Moscow, Russia; 2 Department of Regenerative Medicine, Cell Science Research Center, Royan Institute for Stem Cell Biology and Technology, ACECR, 16635148 Tehran, Iran

**Keywords:** microRNA-mediated regulation, burn wound pathophysiology, mechanisms of burn wound conversion, molecular determinants of burn regeneration, microRNA therapeutics

## Abstract

Burn injury represents a complex trauma involving numerous local and systemic pathological alterations, which are frequently exacerbated by the phenomenon of burn wound conversion or secondary deepening. Conventional clinical management relies heavily on the application of skin grafts, which primarily serve to provide temporary wound coverage and environmental isolation. However, such grafting procedures often prove insufficiently effective and fail to prevent the progression of the burn wound. While contemporary tissue engineering strategies aim to correct specific pathological processes, they fall short of achieving complete and functional restoration of the skin. Consequently, there is a pressing need for the development of novel therapeutic interventions capable of exerting a multifactorial influence on the regeneration process. A promising avenue of research involves microRNA-based therapeutics. These RNA molecules function as master regulators of gene expression, capable of simultaneously modulating entire clusters of genes implicated in wound regeneration and the prevention of burn wound conversion. Therapeutic strategies focused on the targeted delivery of microRNAs to the wound bed hold the potential for creating effective treatment modalities that surpass existing options and effectively prevent burn deepening. This review consolidates current knowledge on the pathogenesis of burn wounds, elucidates the complex interplay between genes and microRNAs during tissue regeneration and describes both existing and experimental microRNA-based delivery approaches aimed at minimizing burn-related complications and enhancing the quality of tissue repair.

## 1. Introduction

The skin serves as the body’s outermost barrier and contains a dense network of sensory nerve endings capable of detecting a wide range of physical and chemical stimuli. It critically governs fluid balance, thermoregulation and metabolic processes, in addition to providing a physical and immunological shield against environmental challenges [1]. Furthermore, beyond its barrier function, the skin is now recognized as the most extensive and immunologically active organ system. Anatomically, the skin comprises three main layers: the epidermis, the dermis and the subcutaneous tissue, also known as the hypodermis. The epidermis comprises a multilayered keratinized squamous epithelium, with keratinocytes accounting for approximately 95% of its cellular constituents. Other resident cell types include melanocytes, Merkel cells, Langerhans cells, CD8+ T cells, corneocytes and stem cells. Between the epidermis and the dermis lies the basement membrane, a specialized fibrous matrix that provides a niche for keratinocyte stem cells, which are essential for maintaining epithelial homeostasis and wound healing [2].

The dermis, located deep under the epidermis, forms a structural scaffold consisting of an extracellular matrix (ECM) and various cell types, including pericytes, endothelial cells, smooth muscle cells, immune cells and fibroblasts [3]. Although fibroblasts represent a numerical minority among these cell types, they play a critical role in skin biology. These multifunctional cells not only synthesize and organize ECM components but also secrete an array of growth factors, cytokines and matrix metalloproteinases (MMPs) that collectively drive the continuous remodeling of the dermal matrix. The ECM itself is principally composed of three key elements: collagen fibers that provide tensile strength, elastin fibers, conferring tissue elasticity and resilience, and proteoglycans for maintaining a hydrated, osmotically active matrix environment that facilitates molecular transport and cellular signaling [4]. The dermal architecture additionally incorporates specialized functional units including vascular networks, sweat glands, hair follicles, neural elements and lymphatic channels.

Due to its barrier function, the skin is constantly exposed to destructive chemical, physical and biological agents. Among these, thermal injuries represent the most prevalent form of trauma with potentially life-threatening consequences. Burns are complex injuries characterized by both local and systemic alterations, which are further exacerbated as the burn depth increases [5]. Thermal wounds rank as the fourth most common traumatic injury worldwide, affecting over 11 million people annually and accounting for an estimated 180,000 deaths worldwide, a mortality rate of around 5% that is comparable to acute myocardial infarction. Data from the Global Burden of Disease study (2021) quantify this burden at an estimated 12.99 million cases of severe burns and 235.34 million mild burns globally. Although prevalence rates have seen a modest decline over the past three decades, the absolute number of cases remains high and is projected to increase, especially in low- and middle-income countries. A particularly vulnerable demographic is children, who suffer a disproportionately high incidence of burns, primarily within the home environment: the mortality rate for children in low- and middle-income countries is currently over seven-fold higher than in high-income ones [6]. Beyond mortality, non-fatal burns are a leading cause of substantial morbidity, entailing prolonged hospitalization, disfigurement and disability [7]. The direct financial cost of burn care is considerable, with estimates ranging from USD 26 to 211 million annually worldwide [8].

Given this significant health and economic burden, the primary objective of burn care is to achieve rapid and effective wound closure. Current burn treatment paradigms primarily involve skin grafts of various biological origins, which serve as temporary barriers to protect the wound from the external environment. Nevertheless, such interventions are still limited by three key factors: marginal therapeutic effectiveness, iatrogenic pain and an inability to halt the dynamic pathophysiological processes of burn progression. A more recent advancement is the development of tissue-engineered constructs, which combine a three-dimensional scaffold with embedded cellular components and essential growth factors to promote tissue regeneration. However, such strategies remain largely symptomatic, aiming to mitigate specific pathological features rather than addressing the complex and multifactorial nature of burn injury [9]. This clinical problem requires the development of therapeutic strategies that can address multiple factors. In this context, microRNA-based therapeutics represent a promising strategy for modulating the pathogenic cascade of burn injury. These small non-coding RNA molecules regulate gene expression across all stages of the pathogenic process, affecting clusters of co-expressed genes whose expression patterns limit complete tissue regeneration. Consequently, developing tailored microRNA therapeutic panels could lead to the creation of significantly more effective treatment modalities compared to current burn therapies [10].

This review will explore the key mechanisms of burn pathogenesis and progression with a particular focus on gene–gene and gene–microRNA interactions involved in the regenerative process. Additionally, we will discuss current therapeutic and experimental approaches utilizing microRNA delivery systems to mitigate burn complications and enhance tissue repair. To date, microRNA expression profiles of burn injury are not uniform but vary according to burn severity. Stratifying patients by wound classification is not only clinically essential for prognosis and treatment planning but also provides a framework for designing microRNA-based diagnostic or therapeutic approaches. In the following section, we therefore outline the conventional clinical and histological classification of burn wounds as a foundation for understanding their diverse molecular pathways and regenerative outcomes.

## 2. Classification of Burn Wounds

**Figure 1:**
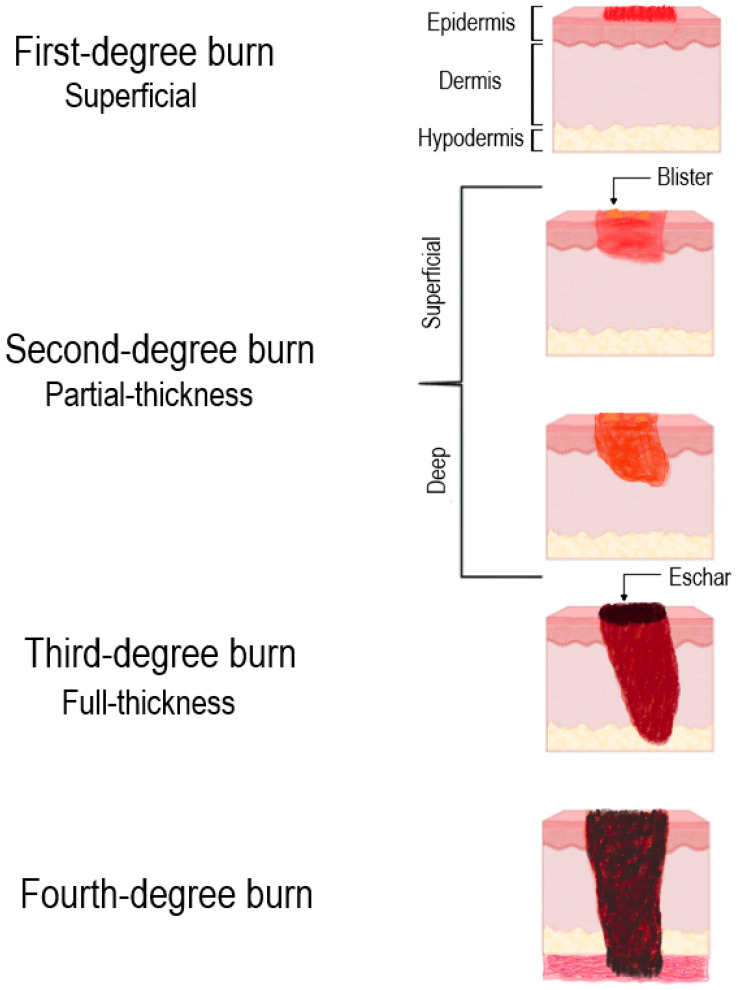
Classification of burn injuries based on the depth of tissue involvement. Illustration generated with Biorender.com.

Burn injuries are classified by the depth of tissue damage, which directly determines their healing potential and risk of pathological outcomes. Burns that involve only the epidermis are classified as superficial (first-degree) burns; they usually resolve within a few days through keratinocyte proliferation without scarring [11]. Partial-thickness (second-degree) burns affect the dermis and are subdivided into superficial and deep types. Superficial partial-thickness burns often heal within three weeks with minimal complications, whereas deep partial-thickness injuries penetrate the reticular dermis, heal slowly and carry a substantial risk of fibrosis and contractures [12]. Full-thickness (third-degree) burns result in complete destruction of the dermis and subcutaneous tissue, forming necrotic eschar and invariably requiring surgical management [13]. The most severe, fourth-degree burns, extend into muscle, tendons, or even bone, with profound systemic consequences and high mortality [14]. Figure 1 provides a schematic overview of the clinical and histological characteristics that define each degree of burn injury.

Severe burn injuries induce profound systemic effects, particularly involving hemodynamic alterations such as vasoconstriction and cardiac dysfunction. These injuries trigger massive protein-rich fluid shifts from the intravascular to interstitial compartments due to increased vascular permeability. Furthermore, they cause substantial bone marrow disturbances, including increased production of immature granulocytes, fragmentation of circulating erythrocytes and neutrophil dysfunction, all of which collectively elevate the risk of sepsis and thrombocytosis [15]. At the cellular level, burn trauma disrupts normal protein homeostasis, inducing widespread protein misfolding and degradation that shifts overall metabolism toward catabolic states. This metabolic dysregulation manifests as systemic insulin resistance, hypermetabolism and dramatic depletion of cellular ATP stores, severely compromising energy-dependent repair processes [16].

## 3. Pathogenetic Aspects of Burn Wound Damage

**Figure 2:**
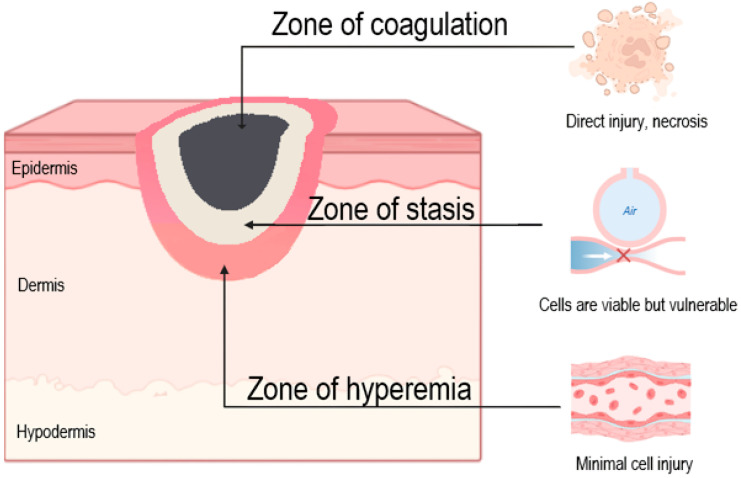
Schematic representation of the classic Jackson zones of burn injury. Illustration generated with Biorender.com.

Burn wounds exhibit three distinct zones of tissue damage, originally described by Jackson in 1953: the zone of coagulation, zone of stasis and zone of hyperemia (Figure 2).

The zone of stasis creates a tissue environment with bipotential outcomes: either recovery or progression to full-thickness necrosis within 48–96 h post-injury [17]. This phenomenon of burn wound conversion involves multiple interconnected pathological mechanisms: sustained ischemia-reperfusion injury, exaggerated inflammatory response, accelerated apoptosis, excessive reactive oxygen species (ROS) production, bacterial colonization and dysregulated autophagy [18]. In the early phase of burn injury, neutrophils infiltrate the interstitial space and release ROS, proteolytic enzymes and other bioactive molecules that contribute to wound progression by damaging adjacent, initially viable tissues [19]. Microthrombosis also develops gradually as red blood cells accumulate in the lumen of small vessels within 2–3 h after injury. These cascades collectively impair tissue oxygenation, promote metabolic acidosis and drive secondary necrosis, explaining why initial clinical assessment often underestimates ultimate burn depth, particularly in intermediate-depth injuries where the zone of stasis predominates [20].

In addition to necrosis, burn injuries induce secondary apoptosis that further contributes to burn wound progression. This programmed cell death is primarily triggered by the pro-inflammatory wound microenvironment, characterized by excessive production of inflammatory mediators, sustained infiltration of neutrophils and M1-polarized macrophages, oxidative stress and impaired tissue perfusion. The resulting apoptotic cascade not only expands the initial injury but also plays a pivotal role in the conversion of partial thickness burns to full thickness lesions [21]. Consequently, therapeutic strategies combining microcirculatory support and controlled immunomodulation may prove critical for preventing secondary burn progression.

The immediate local response to burn trauma involves protein denaturation and release of toxic metabolites, antigens and immunomodulatory agents that collectively contribute to the pathophysiological manifestations of burn shock. Among the earliest and most critical mediators is histamine, which rapidly increases capillary permeability through H1 receptor-mediated contraction of vascular endothelial cells, creating transient intercellular gaps that facilitate plasma extravasation. This process is markedly amplified by the catalytic activity of xanthine oxidase (XO), which not only potentiates histamine’s effects but also generates ROS (hydrogen peroxide and hydroxyl radicals) that directly injure endothelial cells and disrupt collagen cross-linking, further compromising vascular integrity [22]. The resulting burn edema develops through this synergetic interplay of biochemical mediators, including serotonin, bradykinin, nitric oxide, eicosanoids and pro-inflammatory cytokines (tumor necrosis factor-alpha (TNF-α), and interleukins IL-1β, IL-6), which collectively establish a self-perpetuating cycle of vascular leakage and tissue damage. Oxidative stress-induced membrane disruption additionally fuels the release of pro-inflammatory factors, which further dysregulates intercellular junctional integrity and severely compromises fluid homeostasis. This local vascular dysfunction facilitates the infiltration of immune cells into the injured tissue and promotes the systemic dissemination of damage-associated molecular patterns (DAMPs) and inflammatory mediators, thereby contributing to the onset of systemic inflammatory response syndrome (SIRS) and increasing the risk of multi-organ failure. This hyperinflammatory state induces simultaneous immunosuppression, creating the paradoxical clinical picture of systemic inflammation coupled with immunodeficiency that predisposes to sepsis and multi-organ dysfunction [23].

Severe burn injuries induce profound, long-lasting pathophysiological alterations across virtually all organ systems, mediated through interconnected inflammatory, metabolic and endocrine dysregulation. The hepatic response involves significant hepatocyte apoptosis and metabolic dysregulation, contributing to SIRS, while renal manifestations include progressive interstitial edema and tubular degeneration that frequently progresses to acute tubular necrosis. This multi-organ dysfunction is perpetuated by a sustained endocrine stress response characterized by elevated glucocorticoids, glucagon and catecholamines, which synergize with persistently high levels of pro-inflammatory cytokines, reactive oxygen species and nitric oxide to establish a chronic hypermetabolic state that may persist for years post-injury [24]. The clinical consequences of this maladaptive physiology are far-reaching: burn survivors demonstrate markedly increased lifetime risks of cardiovascular disease, metabolic syndrome, diabetes mellitus and musculoskeletal disorders, establishing severe burns as a chronic metabolic condition rather than an isolated acute injury [25]. Persistent elevation of inflammatory mediators (particularly IL-1, IL-6, TNF-α and insulin-like growth factor 1 (IGF-1)) has been mechanistically linked to the maintenance of hypermetabolism in moderate-to-severe burns through both direct and indirect pathways [26]. These cytokines impair myocardial contractile function while simultaneously promoting catabolic bone remodeling via increased osteoclast activity and suppressed osteoblast function, leading to rapid bone mineral density loss [27]. The resulting cardiometabolic–osteoporotic syndrome exemplifies the multisystem nature of post-burn pathophysiology, where chronic inflammation serves as the common mediator connecting apparently disparate complications.

## 4. Skin Regeneration

Under normal physiological conditions, the skin exhibits remarkable regenerative capacity, with acute wounds typically healing spontaneously within 8–12 weeks. Wound repair represents a complex, multifactorial process requiring coordination between diverse cell types (keratinocytes, fibroblasts and endothelial cells), molecular mediators (growth factors, cytokines and chemokines) and the wound bed microenvironment.

**Figure 3:**
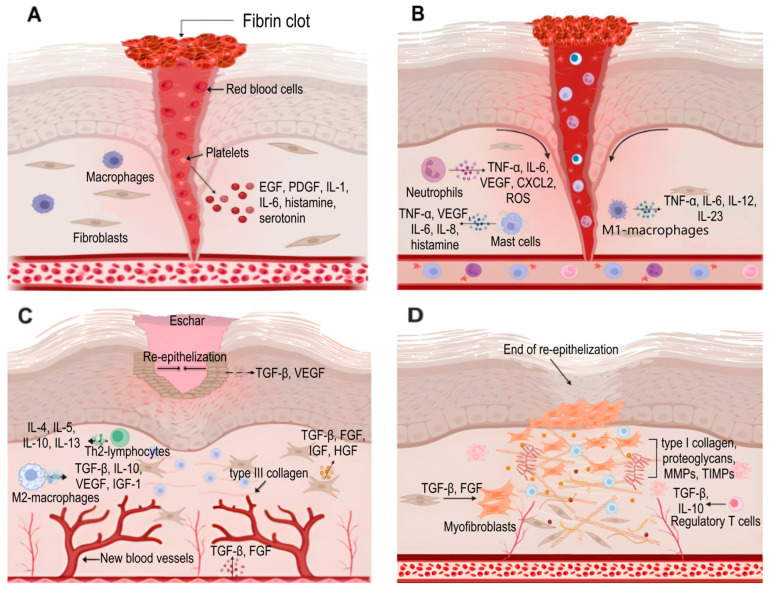
Phases of cutaneous wound healing. (**A**) Hemostasis: Immediate vasoconstriction is followed by platelet aggregation and formation of a fibrin-rich clot, which seals the wound and releases key growth factors and cytokines to initiate the healing cascade. EGF—epidermal growth factor; PDGF—platelet-derived growth factor. (**B**) Inflammation: Neutrophils infiltrate the wound to clear pathogens and debris. They are subsequently replaced by macrophages, which perpetuate the inflammatory response and phagocytose apoptotic cells, transitioning the process to the next phase. VEGF—vascular endothelial growth factor; CXCL2—C-X-C motif chemokine ligand 2. (**C**) Proliferation: Angiogenesis, fibroblast proliferation and deposition of provisional extracellular matrix, and migration of keratinocytes to restore the epidermal barrier (re-epithelialization). TGF-β—transforming growth factor beta; FGF—fibroblast growth factor; HGF—hepatocyte growth factor. (**D**) Remodeling (Maturation): The final, prolonged phase involves the gradual maturation and realignment of collagen fibers (from type III to type I), apoptosis of surplus cells and contraction of the wound. This process results in the replacement of granulation tissue with an acellular scar tissue. TIMPs—tissue inhibitor of metalloproteinases. Illustration generated with Biorender.com.

The skin repair process traditionally includes four phases: hemostasis, inflammation, proliferation and remodeling [28] (Figure 3).

Hemostasis represents the most transient but critical initial stage of wound repair, serving to terminate hemorrhage through a sequence of vascular and cellular responses. Burn wounds present unique hemostatic challenges due to the non-viability of the majority of the injured tissue and its lack of significant blood supply; consequently, blood loss observed in burn injuries is substantially lower compared to other wound types. However, the coagulation of tissue induced by high temperatures results in damage to the vascular network of the burned tissue and an increase in capillary permeability. This leads to alterations in cellular electrolyte composition and the development of ischemia. The initial vasoconstrictive response radiates from the zone of coagulation into the zone of stasis, exacerbating necrotic tissue progression through three key mechanisms: microvascular thrombosis, endothelial dysfunction and impaired oxygen delivery. This pathological cascade not only extends burn depth but also fundamentally compromises tissue regenerative capacity by establishing a pro-fibrotic microenvironment, factors that collectively contribute to prolonged patient recovery timelines and suboptimal functional outcomes [29].

Following hemostasis, the immediate activation of the complement system triggers mast cell degranulation with consequent histamine release, inducing capillary dilation and increased vascular permeability that facilitates the influx of inflammatory cells into the wound bed [30]. Within hours post-burn, severe thermal injury activates the Nuclear factor κB (NF-κB) transcriptional pathway, upregulating TNF-α and intercellular adhesion molecule-1 (ICAM-1) expression, mediators that enhance neutrophil and macrophage antimicrobial activity through phagocytosis of invading pathogens, neutrophil extracellular trap (NET) formation and reactive oxygen species production. The early inflammatory milieu is dominated by M1-polarized macrophages that not only phagocytose bacteria and cellular debris but also secrete pro-inflammatory cytokines (IL-1β, IL-6, IL-12 and TNF-α) and chemokines (C-C motif ligand 2 (CCL2) and CXCL8) to recruit additional leukocytes while initiating granulation tissue formation [31]. This pro-inflammatory state undergoes gradual transition during late inflammation through the coordinated infiltration of T-lymphocytes producing interferon gamma (IFN-γ), TGF-β, IL-4 and IL-10, which drive macrophage polarization toward the anti-inflammatory M2 phenotype essential for the resolution of inflammation and tissue repair. In burn wounds, this transition is frequently delayed or incomplete due to persistent microbial colonization and excessive damage-associated molecular patterns, resulting in chronic inflammation that impairs epithelialization and promotes fibrotic scarring [32].

The proliferative phase emerges approximately 72 h post-injury, marking a transition from inflammation to active tissue reconstruction that typically persists for 2 weeks. The phase progresses rapidly in superficial burns due to the preservation of epidermal stem cells within specialized niches of intact dermal appendages, including the hair follicle bulge, sebaceous gland base and interfollicular epidermal basal layer. In contrast, deep burns extending into the reticular dermis demonstrate markedly delayed healing, as re-epithelialization can only originate from wound margins and cannot commence until necrotic progression is halted [33].

**Figure 4:**
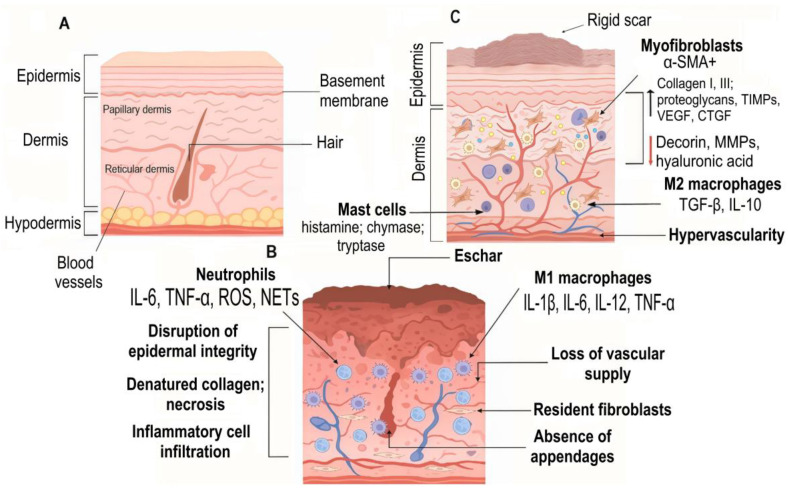
Schematic representation illustrating the histological and cellular alterations observed after burn injury (**B**) compared to normal skin (**A**) and the regenerative phase ending with hypertrophic scar formation (**C**). The black arrow indicates a high level of the listed proteins, while the red arrow indicates a low level of the molecules; CTGF—connective tissue growth factor. Illustration generated with Biorender.com.

The final remodeling phase can persist for months to years after wound closure and involves the gradual transformation of the nascent ECM into mature scar tissue. Concurrent apoptosis of immune cells, endothelial cells, keratinocytes and myofibroblasts determines the final scar architecture. In contrast, burn injuries induce pathological persistence of α-smooth muscle actin (α-SMA)-positive myofibroblasts in scar tissue, driven by sustained TGF-β/Smad signaling. This dysregulation results in excessive extracellular matrix deposition and progressive wound contraction, ultimately leading to fibrotic scarring and contractures (Figure 4) [34].

The resulting scar tissue exhibits permanent loss of cutaneous appendages (hair follicles and sweat glands) and neural elements, manifesting clinically as impaired thermoregulation, sensory deficits and functional limitations, hallmarks of pathological burn wound repair.

## 5. Current Therapeutic Approaches in Burn Management: Challenges and Limitations

Burn injuries represent a persistent clinical challenge, with severe cases characterized by the complete destruction of epidermal and dermal structures, including vascular and neural networks. The therapeutic approach to burn injury management is determined primarily by the depth tissue damage, and the goals of initial treatment for minor burns are to halt the progression of tissue damage, ensure wound cleansing and provide effective analgesia [35].

The management of superficial (first-degree) and superficial partial-thickness (second-degree) burns is conservative. Immediate and sustained cooling with running tap water (8–25 °C) for at least 20 min is a critical first-aid measure proven to reduce burn depth, expedite re-epithelialization and minimize the need for surgical intervention. While superficial burns often heal without specialized dressings, the application of moist gauze can alleviate pain, an effect often supplemented with oral non-steroidal anti-inflammatory drugs [21]. For superficial second-degree burns with the blister skin removed, it is advisable to prioritize the use of biological dressings, such as allogeneic/xenogeneic skin, amniotic membrane or artificial synthetic temporary skin substitutes, for covering the affected area. These materials provide a protective barrier that maintains a moist microenvironment.

Deep partial-thickness burns require a more intensive approach to prevent infection and promote healing in a compromised dermal bed. Management centers on thorough debridement of non-viable tissue, which can be achieved through enzymatic agents (e.g., collagenase and bromelain) or autolytic methods with hydrogels, often followed by the application of advanced dressings. Silver-impregnated foam dressings remain a mainstay in clinical practice due to their potent antimicrobial properties and superior exudate management, which enhances patient comfort by reducing dressing change frequency. In recent years, adjunctive therapies such as autologous platelet-rich plasma (PRP) and bioactive hydrogels have demonstrated promise in accelerating wound closure by mimicking a physiological healing milieu. For deep partial-thickness injuries with a prolonged healing timeline, surgical excision followed by temporary coverage with xenografts or skin substitutes is the standard of care to prevent hypertrophic scarring and contractures [36]. Negative-Pressure Wound Therapy (NPWT) further expands the therapeutic arsenal for complex or highly exudative wounds. By applying controlled sub-atmospheric pressure, NPWT effectively removes exudate, reduces edema and increases local perfusion. At a cellular level, the induced mechanical strain stimulates proliferation and angiogenesis, thereby accelerating granulation tissue formation and epithelialization, while the sealed system minimizes the risk of infection [37].

Full-thickness (third-degree) burns necessitate urgent surgical intervention. The cornerstone of treatment is early excision of the necrotic eschar followed by definitive wound closure. Pending autografting, deep burns are managed with advanced antimicrobial dressings or temporary skin substitutes to reduce pain and fluid loss while allowing wound bed monitoring. Autologous split-thickness skin grafting is the gold standard for definitive wound closure, as it permanently restores the patient’s own epidermis. The success of grafting relies on a well-vascularized wound bed, which can be prepared and protected using NPWT or temporary xenografts. In addition, aggressive fluid resuscitation, nutritional support and systemic antimicrobial therapy are critical components of managing major burn injuries to mitigate systemic inflammatory response and sepsis [12]. The management of fourth-degree burns is primarily surgical and supportive, involving radical debridement, amputation of non-viable extremities and subsequent complex reconstruction using flaps or grafts.

Conventional approaches, while considered the current standard of care, present significant limitations that hinder optimal patient outcomes. Autograft procedures require the creation of additional donor-site wounds, imposing further physiological stress through pain responses and exacerbating fluid/electrolyte imbalances while also being severely constrained by the availability of unaffected skin in patients with extensive burns [38]. Alternative biological grafts carry inherent risks of pathogen transmission, immune rejection and ethical concerns regarding their procurement and use [39]. These shortcomings have driven intensive research into tissue engineering solutions designed to provide physiologically functional skin substitutes that address the fundamental requirements of burn wound management: immediate barrier protection against infection and fluid loss, coupled with long-term promotion of regeneration rather than scarring. Contemporary engineered constructs combine biocompatible scaffolds with bioactive molecules and cellular components to create immunologically compatible wound coverings that have demonstrated clinically significant improvements in epithelialization, scar quality and functional outcomes. The most promising developments in this field include three-dimensional bioprinted autologous skin equivalents with precise architectural control, smart matrices incorporating controlled-release bioactive molecules payloads targeting fibrotic pathways and bioactive dressings capable of dynamic interaction with the wound microenvironment [40]. Table 1 summarizes principal wound-care modalities currently used in clinical burn management, organized by therapeutic class.

Despite significant advances in burn care, current therapeutic platforms often fail to achieve complete functional tissue restoration, frequently resulting in chronic pathological progression. Addressing this clinical challenge requires molecular-level interventions utilizing gene therapy and gene engineering approaches. While delivery of bioactive molecules (DNA, mRNA or small interfering RNAs (siRNAs)) via viral or non-viral vectors can modulate cellular processes to enhance skin regeneration, the most transformative opportunity lies in small non-coding RNA therapeutics, particularly microRNAs (miRNAs) that directly regulate protein synthesis through post-transcriptional gene silencing [50]. It is well established that the miRNA expression profile is dynamically altered in various pathological states: some miRNAs become overexpressed, while others are underexpressed, creating a distinct, disease-specific miRNA signature within the cell. This dysregulation presents a therapeutic opportunity to correct the aberrant expression profile through the administration of synthetic miRNA inhibitors (antagomiRs) or mimics. The introduction of antagomiRs effectively knocks down excessive levels of endogenous miRNAs, thereby de-repressing their target genes and restoring normal protein expression. In contrast, replacement therapy with synthetic miRNA mimics is designed to restore the function of deficient miRNAs. While this does not always increase the transcriptional expression of the endogenous miRNA itself, it potently amplifies its functional activity by supplying the cell with mature, synthetic duplexes that mediate interactions with the necessary downstream gene targets [51]. By modulating key miRNAs involved in inflammation, fibrosis and angiogenesis, it becomes possible to intervene at multiple stages of wound healing. These approaches offer precise control over the wound microenvironment, addressing the root causes of pathological healing rather than just symptoms [50].

## 6. Role of MicroRNAs in Burn Wound Pathophysiology

MicroRNAs (miRNAs) represent a class of highly conserved, small non-coding RNAs ranging from 19 to 25 nucleotides in length. Since their discovery, miRNAs have emerged as pivotal regulators of gene expression and modulate the expression of nearly 60% of human genes at both translational and post-translational levels [52]. The canonical mechanism of miRNA action involves the binding of the mature miRNA to complementary sequences within the 3′-untranslated region of target messenger RNAs (mRNAs). This interaction typically results in deadenylation, removal of the 5′-cap structure, translational repression and, eventually, the degradation of the target transcript.

**Figure 5:**
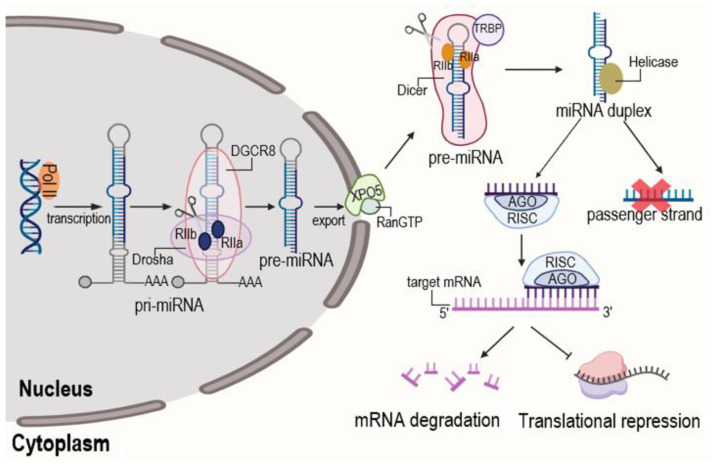
The canonical pathway of microRNA biogenesis and function. Illustration generated with Biorender.com.

MiRNAs are transcribed by RNA polymerase II (Pol II) as long primary transcripts (pri-miRNAs) that form hairpin structures. The microprocessor complex, comprising the RNase III enzyme Drosha and its cofactor microprocessor complex subunit DGCR8, cleaves the pri-miRNA in the nucleus to release a shorter hairpin precursor (pre-miRNA). This pre-miRNA is exported to the cytoplasm by Exportin-5 (XPO5)-Ran-GTP. In the cytoplasm, the RNase III enzyme Dicer, in complex with TRBP subunit of RISC loading complex, cleaves the pre-miRNA loop to generate a transient miRNA duplex. This duplex is loaded into the Argonaute (AGO) protein within the RNA-induced silencing complex (RISC). The guide strand (mature miRNA) is retained, while the passenger strand is typically degraded. The mature RISC then mediates post-transcriptional gene silencing by binding to complementary sequences on target mRNAs, leading to translational repression or mRNA deadenylation and decay [53] (Figure 5).

While numerous protein biomarkers have been investigated for assessing burn pathology and guiding treatment decisions, their clinical application faces some limitations, including insufficient sensitivity for early depth assessment, low specificity in distinguishing between burn severities and unreliable prognostic value for healing outcomes. These disadvantages highlight the urgent need for alternative molecular markers capable of providing accurate, non-invasive evaluation of burn severity while simultaneously serving as therapeutic targets [54]. Circulating microRNAs have emerged as particularly promising candidates, offering three advantages over conventional protein biomarkers: their high specificity through unique expression signatures corresponding to specific injury, high sensitivity and ease of detection in minimally invasive samples such as plasma, serum and wound exudate. Beyond their diagnostic utility, miRNA-based therapeutics offer opportunities through the restorative strategies involving administration of depleted miRNAs and inhibitory approaches utilizing normalization of overexpressed miRNAs. This dual diagnostic–therapeutic potential positions miRNAs as versatile tools for personalized burn management, enabling early severity stratification through specific miRNA signatures followed by modulation of healing pathways [55].

MicroRNAs demonstrate phase-specific expression patterns and functional specialization throughout the burn healing process, orchestrating complex gene regulatory networks at each stage of repair (Table 2).

The hemostasis phase involves an orchestrated microRNA-mediated regulation of vascular and coagulation responses. The miR-143/145 cluster plays a pivotal role in modulating vascular dynamics, facilitating the transition from initial vasoconstriction to vasodilation in the wound bed by targeting key regulators of vascular tone [56]. Concurrently, fibrinogen synthesis is regulated by miR-409-3p and members of the miR-29 family, which target the 3′-untranslated regions of fibrinogen genes (*FGA*, *FGB* and *FGG*). This regulation ensures a balanced fibrin formation: sufficient to establish initial clot stability while preventing excessive thrombosis that could compromise microcirculation in the vulnerable zone of stasis [59].

The impact of burn injury on physiological homeostasis is reflected in platelet dynamics, which typically show a nadir around day 3 post-burn, followed by a rebound thrombopoietic phase peaking near day 15 before gradual normalization. The therapeutic application of platelet-rich plasma, rich in growth factors (PDGF, TGF-β, FGF, EGF and VEGF) and microRNAs, has been shown to accelerate closure even in deep burns, reduce cell death and attenuate neutrophil infiltration in the zone of stasis [90]. Activated platelets release a diverse repertoire of microRNAs—most abundantly miR-223, miR-21-5p, miR-23a-3p and miR-126-3p—that are directly implicated in hemostasis, thrombosis and regeneration. For instance, lower expression of miR-223 may increase platelet reactivity and ensure effective clot formation [91], while miR-126 overexpression supports vascular integrity and endothelial repair and enhances thrombus formation [92]. Beyond hemostasis, these miRNAs are key modulators of the ensuing inflammatory and angiogenic phases, a role detailed later in this review.

During the inflammatory phase, the first line of defense is the innate immune system, which is activated in response to damage to the skin barrier. Neutrophils, the first responders, are recruited to perform phagocytosis, oxidative burst and release neutrophil extracellular traps. Their function is tuned by specific miRNAs: miR-142-3p enhances neutrophil chemotaxis and polarization by affecting GTPase translation, thereby influencing phagocytic capacity and reactive oxygen species production [93]. The activation of neutrophils by various inflammatory stimuli induces the release of extracellular vesicles that are internalized by endothelial cells (ECs), leading to the transfer of miRNAs including miR-223, miR-142-3p and miR-451 and consequent endothelial damage. While miR-223 exerts minimal effects on EC function, the induced expression of miR-142-3p and miR-451 profoundly compromises endothelial integrity, particularly under inflammatory conditions, resulting in increased apoptosis, impaired angiogenic repair and upregulation of pro-inflammatory cytokines and chemokines such as IL-6, IL-8, CXCL10 and CXCL11. Mechanistically, the deleterious impact of miR-142-3p may be mediated by its inhibition of extracellular signal-related kinases 1 and 2 (ERK1/2) and endothelial nitric oxide synthase (eNOS) signaling, as well as the suppression of key genes involved in EC migration and angiogenesis, including Rac family small GTPase 1 (RAC1), Rho associated coiled-coil containing protein kinase 2 (ROCK2) and chloride intracellular channel 4 (CLIC4) [94].

MiR-223 serves as a key anti-inflammatory regulator, exerting pleiotropic control over neutrophil biology. It directly suppresses the transcription factor MEF2C (myocyte enhancer factor 2 C), thereby modulating neutrophil differentiation, survival and functional activation [95]. A reduction in miR-223 expression at the site of injury leads to a hyperactive neutrophil phenotype, characterized by excessive infiltration and prolonged inflammation [96]. Furthermore, miR-223 resolves neutrophil-mediated inflammation and facilitates healing by dampening the classical NF-κB pathway through direct targeting of adaptor protein TNF receptor-associated factor 6 (TRAF6), effectively reducing NF-κB activation and suppressing the inflammatory cascade [95].

Subsequent to neutrophils, macrophages are recruited to coordinate inflammation, regeneration and antimicrobial defense [26]. Their recruitment and polarization are critically influenced by microRNAs. MiR-124a regulates monocyte migration by targeting ICAM-1 and CCL2 [97], while activation of the M-CSFR gene occurs via miR-424-mediated suppression of NFI-A translation [61]. Within macrophages, miR-21 enhances the efferocytosis of apoptotic cells and exerts a potent anti-inflammatory effect by stimulating c-Jun-AP-1 activity, which consequently inhibits NF-κB activation and TNF-α expression while enhancing IL-10 production [62]. Similarly, miR-146a is renowned for its anti-inflammatory and pro-angiogenic properties [98], achieved by targeting key kinases in the NF-κB pathway (interleukin-1 receptor-associated kinase 1 and 2 (IRAK1, IRAK2) and TRAF6) in monocytes, macrophages and keratinocytes, thereby enhancing VEGF-A expression. It also modulates apoptosis by reducing Bcl-2 protein expression and curbing IL-6 production [99]. In contrast, miR-218-5p promotes apoptosis by targeting the Wnt pathway activator SFRP2 (secreted frizzled-related protein 2); its local suppression can thus serve as a protective mechanism against excessive cell death [24]. Another critical regulator, miR-132, limits pro-inflammatory cytokine overproduction via NF-κB suppression. Its expression increases during the transition to the proliferative phase, where it stimulates keratinocyte proliferation through signal transducer and activator of transcription 3 (STAT3) and mitogen-activated protein kinase (MAPK) pathway activation. Inhibition of miR-132 leads to severe inflammation, reduced keratinocyte growth and significantly delayed regeneration [66].

The regenerative outcomes following burn injury vary depending on burn depth and severity. In cases of deep but localized injuries, the healing process occurs primarily through wound contraction and keratinocytes migration from the wound margins. These cells exhibit upregulated expression of miR-31 to promote proliferation and migration by targeting epithelial membrane protein 1 (EMP-1), which helps restore skin barrier function [71]. A natural inhibitor of this migratory process is miR-198, which is transcribed from the 3′-untranslated region of follistatin-like protein 1 (FSTL1) mRNA, a protein that itself facilitates keratinocyte migration. Under normal regenerative conditions, the expression of the splicing regulator protein K-homology type (KSRP), responsible for miR-198 processing, is suppressed. This suppression reduces miR-198 levels, allowing increased FSTL1 production and enhanced keratinocyte migration. However, in chronic wounds, miR-198 expression remains elevated, impairing cell migration, inhibiting re-epithelialization and promoting the transition from acute to chronic wound states [72].

Similarly, miR-21 and miR-130a demonstrate complex roles in epithelialization. In human venous trophic ulcer models, their overexpression has been associated with delayed re-epithelialization [76]. Paradoxically, knockdown of miR-21 also reduces keratinocyte and fibroblast migration, disrupting re-epithelialization. This dual role appears mediated through miR-21 targeting of inhibitory Smad7; excessive Smad7 expression reduces elastin production via the Smad7-Smad2/3-elastin pathway, ultimately impairing healing [100].

Members of the microRNA-99 family (miR-99a, miR-99b and miR-100) play crucial roles in coordinating keratinocyte behavior during re-epithelialization. Their expression is significantly downregulated in keratinocytes at the wound bed, leading to increased expression of their target genes, insulin-like growth factor 1 receptor (IGF1R) and AKT serine/threonine kinase 1. This derepression enhances both proliferative and migratory capacities of keratinocytes through the activation of the phosphoinositide 3-kinase (PI3K)/AKT signaling pathway, which promotes cell cycle progression and cytoskeletal reorganization necessary for efficient wound closure [73]. Similarly, miR-203 exhibits spatial regulation during healing, with high expression maintained in suprabasal epidermal layers surrounding the wound but minimal levels in actively migrating keratinocytes at the leading edge. This differential expression pattern facilitates its distinct functions: miR-203 negatively regulates two key targets involved in cytoskeletal dynamics: RAN (a small GTPase critical for mitotic spindle assembly) and RAPH1 (a Ras-associated protein involved in actin cytoskeleton reorganization during cell migration). Through the inhibition of these proteins, miR-203 precisely controls the transition between proliferative and migratory states in keratinocytes [77].

The process of re-epithelialization and angiogenesis is finely regulated by multiple microRNAs through distinct mechanistic pathways. miR-378a has emerged as a negative regulator of wound healing by directly targeting vimentin and β3-integrin, two proteins essential for cell migration and adhesion. Experimental knockdown of miR-378a enhances the synthesis of these target proteins, accelerates fibroblast migration and differentiation in vitro and significantly improves wound healing in murine models in vivo. Concurrently, elevated vimentin and β3-integrin levels promote VEGF-mediated angiogenesis, positioning miR-378a as a master regulator of genes associated with cell migration, differentiation and vascular formation. This regulatory function makes miR-378a a promising target for anti-angiogenic therapies, particularly in preventing excessive vascularization that can lead to hypergranulation, fibrosis or keloid scarring [101].

Angiogenesis itself is controlled by a complex network of pro- and anti-angiogenic miRNAs. Pro-angiogenic miRNAs include miR-126, miR-130a, miR-210, miR-23, miR-27 and miR-24, which enhance vascular formation through various pathways [102]. Among them, miR-126 downregulates SPRED1 (sprouty-related EVH1 domain containing 1) and PIK3R2 (phosphoinositide-3-kinase regulatory subunit 2) and enhances VEGF and ANGPT-1 (angiopoietin-1) signaling, thereby coordinating both the initiation and maturation of the vascular network [103]. While VEGF is indispensable for the formation of the primary vascular plexus, ANGPT-1 is required for the stabilization and maturation of developing vessels [104]. Furthermore, miR-126 sustains the proliferation, migration and endothelial differentiation of endothelial progenitor cells (EPCs) while simultaneously preventing their drift toward the hematopoietic lineage through inhibition of HOXA9 (homeobox A9) and blocking the TGFβ-induced mesenchymal transition of EPCs [103].

Conversely, miR-92a, miR-217, miR-221, miR-222 and miR-199a-5p inhibit angiogenesis [102]. Specifically, miR-199a-5p suppresses angiogenesis by targeting transcription factor ETS1 and matrix metalloproteinase MMP1, and its expression is downregulated in dermal and endothelial tissues during regeneration [75]. Similarly, miR-200b exerts anti-angiogenic effects by inhibiting ETS1, GATA binding protein 2 (GATA2) and vascular endothelial growth factor receptor 2 (VEGFR2) [105]. MiR-92a has been characterized as a pivotal regulator of vascular smooth muscle cell (VSMC) proliferation and migration, exerting its effects via signaling cascade involving ROCK/STAT3 and myosin light-chain kinase (MLCK)/STAT3. The activation of these kinases enhances miR-92a expression, which, in turn, suppresses the transcription factor KLF4—an established inhibitor of VSMC growth—thereby promoting PDGF-BB-mediated phenotypic activation of VSMCs. Notably, pharmacological or genetic inhibition of ROCK or MLCK elevates KLF4 levels and impairs VSMC activity, demonstrating that miR-92a serves as a critical downstream effector in this axis [106]. In a complementary context, recent evidence implicates miR-223-3p in the modulation of burn wound healing. Its expression is markedly elevated in patients with deep second-degree burns but decreases significantly by the 28th day of recovery. The transcription factor FOXO1 (forkhead box protein O1) has been identified as a direct target of miR-223-3p, with their expression levels showing a significant inverse correlation: elevated miR-223-3p corresponds to reduced FOXO1 expression. Functional assays further revealed that miR-223-3p overexpression suppresses the viability of human umbilical vein endothelial cells (HUVECs), while the restoration of FOXO1 partially reverses this inhibitory effect. Collectively, these findings suggest that miR-223-3p is closely associated with the progression and resolution of burn injuries and may influence vascular repair by modulating the FOXO1 pathway [107].

The remodeling phase of wound healing is regulated by the miR-29 family. These microRNAs have conserved seed sequences that are complementary to binding sites on collagen-encoding genes, allowing for coordinated control of extracellular matrix remodeling [108]. miR-29a expression is transiently elevated in fibroblasts of denatured dermis shortly after thermal injury, followed by its gradual decline to enhance the expression of target genes *COL1A2* (collagen type I alpha 2) and *VEGF-A*, thereby promoting fibroblast proliferation, migration and modulation of contractility. This regulation is particularly significant given the central role of TGF-β1—present at high concentrations in wound granulation tissue—in stimulating fibroblast-to-myofibroblast differentiation. MiR-29a exerts potent anti-fibrotic effects by inhibiting TGF-β/Smad3 signaling cascades, reducing α-SMA and collagen I/III synthesis in scar tissues while simultaneously increasing MMP1 synthesis to initiate degradation of excess collagen. Additionally, miR-29a regulates skin regeneration through modulation of the Notch/Jagged pathway, which maintains skin homeostasis by mediating fibroblast migration for ECM synthesis and endothelial cell function for angiogenesis [109]. Similarly, miR-29b expression significantly decreases post-burn, leading to increased HSP47 activity and enhanced collagen biosynthesis. HSP47 facilitates proper protein folding through transient interactions with procollagen in the endoplasmic reticulum, and its direct binding to procollagens is critical for subsequent assembly, secretion, cleavage and mature fibril formation [86].

Conversely, miR-145 promotes myofibroblast differentiation and functionality induced by TGF-β1. In vivo, both miR-145 and α-SMA levels are significantly elevated in hypertrophic scars compared to normal skin. miR-145 interacts with the transcription factor KLF4 in fibroblasts, suppressing its expression. Since KLF4 acts as a negative regulator of α-SMA expression and fibroblast-to-myofibroblast differentiation, miR-145-mediated inhibition of KLF4 enhances myofibroblast differentiation and contributes to pathological contracture formation [87].

**Figure 6:**
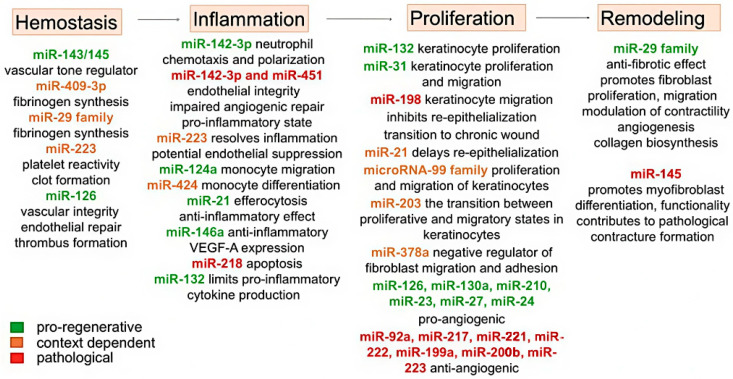
An integrative overview of miRNA involvement in burn wound healing.

The overall interaction between miRNAs during burn wound healing is summarized in Figure 6.

## 7. MicroRNAs and Scar Formation

Pathological scarring remains the most prevalent and difficult-to-treat complication of burn trauma in dermatologic and reconstructive surgery, underscoring the imperative to delineate molecular mechanisms that mitigate its onset [110]. Deep burns frequently result in damage to the reticular dermis accompanied by retained necrotic tissue, which exacerbates the overall tissue damage. Current debridement strategies, ranging from surgical excision to enzymatic and autolytic techniques, are essential for removing necrotic eschar. However, aggressive surgical intervention can cause iatrogenic tissue damage, while enzymatic and other non-surgical methods may yield incomplete necrosectomy. Both scenarios can predispose the wound bed to secondary infection and perpetuate a protracted inflammatory phase that drives fibrogenesis [111].

The pathogenesis of scar formation is fundamentally driven by the dysregulated proliferation of fibroblasts and their pathological overproduction of ECM constituents, notably collagens. Over recent decades, it has been established that although fibroblasts isolated from keloid tissue exhibit a typical spindle-shaped morphology, their gene expression profile and functional behavior are distinct, warranting their classification as keloid-derived fibroblasts (KFs). These cells overproduce ECM components, display accelerated proliferation rates, exhibit enhanced migratory capacity and possess a spectrum of metabolic features similar to tumor cells [112].

A growing body of evidence indicates that microRNAs are key regulators of the biological processes governing the proliferation, differentiation and apoptosis of keloid fibroblasts, with their own expression being significantly dysregulated in fibrotic tissue. For instance, miR-181a is overexpressed in keloid tissue to promote cell proliferation and inhibit apoptosis through suppressing PHLPP2 (PH domain and leucine-rich repeat protein Phosphatase 2) expression, leading to activation of the AKT pathway and, consequently, accelerated cell growth of keloid fibroblasts [113]. Another member of the miR-181 family, miR-181b-5p, exhibits pro-fibrotic activity in hypertrophic scars by potentiating fibroblast proliferation and inhibiting apoptosis through the direct modulation of the MEK/ERK signaling cascade and its downstream effector, the cell cycle inhibitor p21 [114]. Of particular significance is the dysregulation of miR-497-5p, a potent regulator of fibroblast viability. Its expression is markedly elevated in hypertrophic scar tissue while being significantly suppressed in acute burn wound tissue. This miRNA exerts its pro-fibrotic effects through the intricate modulation of key signaling pathways, including NF-κB, FGF-2 and Smad7 [115].

Re-epithelialization is a critical factor of successful wound healing: a skin defect cannot be considered healed until a functional epidermal barrier has been restored, regardless of the underlying structure of the dermis. Emerging evidence over recent years has established that the in vivo administration of specific miRNAs can significantly reduce wound area by stimulating re-epithelialization, recruiting myofibroblasts and enhancing collagen deposition. For instance, expression levels of miR-506-3p and miR-155 are significantly downregulated in fibroblasts isolated from burn tissue compared to intact skin, suggesting their involvement in pathological remodeling and scarring. Experimental transfection of fibroblast cultures with mimics of these miRNAs induced a dose-dependent reduction in cell viability, suppressed hyperproliferation and inhibited the expression of key pro-inflammatory mediators TNF-α, IL-6 and IL-8, culminating in the formation of a more organized regenerated tissue architecture [116]. Furthermore, miR-506-3p modulates fibroblast autophagic activity by directly targeting and inhibiting the synthesis of Beclin-1, thereby constraining excessive collagen production, and mitigating the development of hypertrophic scars [117].

Keratinocytes, the principal cellular constituents of the epidermis, are active participants in wound contraction alongside fibroblast. Particular attention has been focused on miR-21, one of the most extensively characterized miRNA regulators of skin repair. Its expression is markedly elevated in both burn-related and non-burn hypertrophic scars. Functionally, miR-21 enhances keratinocyte migration (including TGF-β1-induced migration), promotes fibroblast proliferation, reduces fibroblast apoptosis and elevates fibroproliferative marker expression (COL1A1, COL3A1 and α-SMA) [118]. These effects are mediated through the targeting of TIMP3 and TIAM (T-lymphoma invasion and metastasis-inducing protein), whose suppression facilitates enhanced cell motility. Additionally, miR-21 regulates hypertrophic scar fibroblast proliferation via the PI3K/AKT pathway by inhibiting phosphatase and tensin homolog (PTEN) and activating telomerase reverse transcriptase (hTERT) [119].

Another significant mediator is miR-31, which also potently enhances keratinocyte proliferation and migration. Real-time PCR analysis revealed increased expression of the proliferation marker Ki-67 in keratinocytes upon miR-31 overexpression [71]. Its functional role was further corroborated by experiments showing that transfection of primary human keratinocytes with pre-miR-31 or an miR-31 mimic significantly enhanced their proliferative capacity compared to cells treated with an miR-31 inhibitor or negative controls [120]. The pro-proliferative effects of miR-31 are partially mediated through the direct targeting and suppression of EMP-1 synthesis [71].

Finally, an important impediment to the healing of chronic wounds is hypoxia. Compromised oxygen delivery triggers the activation of specific “hypoxamiRs,” most notably miR-210. HypoxamiRs are a specific class of microRNAs whose expression is directly induced or suppressed under conditions of low oxygen. They function as crucial molecular switches that help cells adapt and survive in a low-oxygen environment [121]. Under the transcriptional control of HIF-1α/2α, miR-210 represses the expression of E2F3, a transcription factor essential for keratinocyte proliferation, and the iron–sulfur cluster scaffold proteins (ISCU1/2), which are critical for mitochondrial metabolism and Fe-S cluster biogenesis [122]. Suppression disrupts cellular energy homeostasis and creates a significant barrier to effective re-epithelialization under conditions of chronic ischemia [123].

In summary, this collective evidence firmly establishes miRNAs as pivotal regulators of skin cell proliferation, migration and differentiation. Their dysregulation in the context of burn injury and chronic ischemia is a key driver of pathological scarring. Consequently, the targeted modulation of specific miRNA activities—such as miR-21, miR-31, miR-210 and miR-29—represents a highly promising strategic avenue for the molecular therapy of burn and chronic wounds [10].

## 8. MicroRNA-Based Therapeutic Strategies for Mitigating Burn Injury Pathogenesis

The formidable challenge in severe burn management lies in counteracting the pervasive systemic hypermetabolic and hyperinflammatory response, a maladaptive physiological state that propagates secondary tissue injury and perpetuates a cycle of local damage and impaired regeneration. The complications arising from this immune dysregulation, notably septic shock and multiple organ dysfunction syndrome, remain leading causes of mortality in burn patients despite considerable advances in antibiotic therapies and critical care support [124]. Consequently, the active resolution of inflammation within the wound bed presents a critical therapeutic avenue for interrupting this pathogenic cascade and minimizing secondary progression.

Compelling evidence from preclinical models underscores the therapeutic potential of specific microRNAs in modulating the post-burn inflammatory landscape. A representative study investigating the efficacy of miR-181c, delivered via exosomes derived from human umbilical cord mesenchymal stem cells (hUCMSCs), demonstrated profound anti-inflammatory effects in a rat model of third-degree burns. This was evidenced by a sharp reduction in total leukocyte count, alongside decreased levels of key inflammatory mediators TLR4, NF-κB/p65, TNF-α and IL-1β and a concurrent elevation of the anti-inflammatory cytokine IL-10 compared to controls. Histopathological analysis corroborated these findings, revealing a marked attenuation of neutrophil and macrophage infiltration within the wound bed of the treatment group. The proposed mechanism involves the direct targeting of TLR4 mRNA in macrophages by miR-181c, thereby blunting the primary signaling cascade responsible for pro-inflammatory activation [125]. Some miRNAs are indispensable for physiological repair, and their inhibition can be detrimental. Experimental knockdown of miR-147 using LNA-based inhibitors resulted in amplified concentrations of TNF-α and IL-6, establishing its role as a negative feedback regulator of TLR-induced inflammatory responses in macrophages. This endogenous function is critical for preventing the aberrant, protracted inflammation that fuels fibrotic tissue remodeling and scar pathogenesis [63].

MiR-132 was required for normal skin wound healing, because inhibition or knockout with an miR-132-specific, LNA-modified probe in mouse skin led to delayed wound closure accompanied by increased inflammation [66]. Its knockdown precipitates a hyperinflammatory wound milieu, impairs keratinocyte proliferation and migration and significantly prolongs the healing timeline. The therapeutic potential of miR-132 was further validated in a human ex vivo wound model. Liposome-formulated miR-132 mimics or control oligonucleotides were delivered topically using a thermo-reversible Pluronic F-127 gel. As a result, wounds receiving miR-132 mimics were completely re-epithelialized, whereas wounds treated with control oligonucleotides were only partially covered by the newly formed epithelium on day 5 after injury. Moreover, this accelerated healing was corroborated by a significant increase in Ki-67-positive proliferating cells within the wound edge epidermis of miR-132-treated samples [126].

The mechanism of miR-132 is pleiotropic, orchestrating healing across multiple cell lineages relevant to wound repair. When delivered in a neutral lipid emulsion, it simultaneously modulates keratinocytes, macrophages, neutrophils and endothelial cells. During inflammation, it suppresses the expression of pro-inflammatory mediators across these types of cells and is a potent driver of macrophage polarization towards the pro-reparative M2 phenotype, which enhances immunosuppressive functions and promotes tissue regeneration [67]. Its enhanced expression directly augments endothelial cell proliferation and fosters robust angiogenesis, which is critical for restoring nutrient and oxygen supply to the healing tissue [126].

MiR-155 exemplifies the context-dependent duality of miRNA function in wound healing, playing distinct and often opposing roles across different phases of repair. This pluripotent molecule is involved in immune cell development and function, as well as in the establishment of normal tissue architecture during healing. Initially activated during the inflammatory phase, miR-155 exhibits pro-inflammatory properties. Consequently, therapeutic intervention with miR-155-specific antagomir in murine wound models attenuated the recruitment of inflammatory cells to the wound site and subsequently improved the histological architecture of regenerated tissue [127].

The paradigm of therapeutic inhibition extends to other miRNAs. For example, experimental administration of miR-27b inhibitors markedly improved the healing rate of burn wounds in rats (second-degree burn). Specifically, inhibition of miR-27b on day 21 post-burn significantly increased fibroblast density and the expression of key tissue remodeling proteins—collagen I and III, MMP-1 and α-SMA—at the wound site [68]. This was accompanied by reduced inflammatory cell infiltration and improved regenerated tissue morphology. In contrast, wounds treated with an miR-27b mimic exhibited pronounced infiltration and diminished new collagen synthesis and aberrant collagen fiber organization [128].

The clinical translation of RNA interference (RNAi) therapeutics is critically dependent on overcoming the barrier of safe and effective delivery to target tissues. In response, innovative delivery platforms are being engineered. A prominent example is the development of a polyethyleneimine–deoxycholic acid (PEI–DA) copolymer, a modified cationic polymer capable of forming stable polyplex nanoparticles with an LNA-modified miR-92a inhibitor. This system was readily internalized by human endothelial cells without appreciable cytotoxicity and effectively suppressed miR-92a, resulting in upregulation of key angiogenic regulators such as integrin α5 (ITGA5), Sirtuin 1 (SIRT1) and KLF2/4, as well as enhanced endothelial migration and capillary-like tube formation in vitro. When applied in the chick chorioallantoic membrane model and delivered in vivo using a biodegradable polyethylene glycol (PEG) hydrogel for sustained release, the complexes significantly promoted vascular network formation. This hydrogel-mediated delivery system thus demonstrates both efficacy and safety, offering a promising RNAi-based therapeutic platform for localized induction of angiogenesis and improved tissue repair [129].

Notably, the functional role of miRNAs can reverse between healing phases. While miR-155 inhibition is beneficial early on, its exogenous supplementation later, during the scarring, proved advantageous. The introduction of an miR-155 mimic in a rabbit ear scar model significantly reduced wound volume after six weeks compared to controls and promoted a more organized scar texture [130]. Similarly, both miR-155 and miR-506-3p show significantly downregulated expression in fibroblasts from burned human skin, suggesting their involvement in scar pathogenesis. Experimental intervention using artificial skin membrane resulted in a marked upregulation of both miRNAs, concomitant with an improvement in patient rehabilitation outcomes. Mechanistic studies demonstrated that the transfection of fibroblasts with miR-506-3p and miR-155 mimics significantly suppressed cell viability in a dose-dependent manner, indicating that these miRNAs act to inhibit excessive fibroblast proliferation, a key driver of fibrotic tissue formation. Furthermore, this miRNA-mediated suppression of fibroblast hyperproliferation was associated with a substantial decrease in the secretion of TNF-α, IL-6 and IL-8. A key molecular mechanism underlying these observations involves direct targeting of the PIK3CA 3′-untranslated region by both miRNAs. PIK3CA encodes the p110α catalytic subunit of PI3K, a crucial component of the PI3K-Akt signaling pathway [131]. Through this targeting, miR-155 and miR-506-3p suppress PI3K-Akt activation, thereby modulating fibroblast survival, migration, proliferation and collagen production. These findings posit a model wherein the upregulation of miR-155 and miR-506-3p, facilitated by artificial skin membrane treatment, contributes to scar mitigation through direct inhibition of fibroblast hyperproliferation and suppression of a persistent pro-inflammatory microenvironment [116].

Beyond these, other miRNAs present attractive therapeutic targets. For instance, experimental inhibition of miR-145 expression in dermal myofibroblasts induced a substantial upregulation of KLF4 and a concomitant downregulation of α-SMA. This resulted in the suppression of core myofibroblast functions—contractility, ECM component production and TGF-β1 secretion—which act synergistically in hypertrophic scar formation. Therefore, therapeutic inhibition of miR-145 may promote favorable tissue remodeling without pathological contractures, helping to preserve skin functionality [87].

In recent years, extracellular vesicles (EVs) have emerged as promising therapeutic agents in regenerative medicine, particularly for burn wound management and pathological scar prevention. These heterogeneous nanoscale phospholipid bilayer structures—comprising exosomes and microvesicles—function as intercellular communication vehicles. They facilitate the horizontal transfer of bioactive cargo including microRNAs, mRNAs, proteins and lipids to recipient cells, thereby orchestrating complex wound healing processes through epigenetic reprogramming and post-transcriptional regulation [132]. The molecular composition of EV cargo is regulated by cellular activation states and microenvironmental cues, making them ideal biomarkers and therapeutic vehicles for pathological wound conditions.

Mesenchymal stem cell-derived EVs have demonstrated exceptional capabilities in modulating the burn wound microenvironment through multi-modal mechanisms. Their efficacy stems from a complex miRNA repertoire that simultaneously targets inflammatory signaling, angiogenesis and fibrotic pathways [133]. In full-thickness excision models, umbilical cord blood MSC-derived exosomes containing miR-21, miR-23a, miR-125b and miR-145 exhibit potent anti-fibrotic effects through coordinated inhibition of the TGF-β2/SMAD2 pathway [134]. The miR-21-3p particularly enhances re-epithelialization through PTEN inhibition, activating AKT-mediated keratinocyte migration while simultaneously promoting angiogenesis via protein sprouty homolog 1 (SPRY1) suppression [135]. Exosomal microRNAs indeed represent a groundbreaking approach for preventing pathological scarring during wound healing, offering unprecedented precision in modulating the complex molecular networks that govern tissue repair. The safety and efficacy of exosomal therapies, particularly in the context of scar prevention, have been increasingly validated in clinical settings [136].

The translational potential of EV therapies is further enhanced through innovative sourcing strategies. Keratinocyte-derived exosomes from induced pluripotent stem cells (iPSC-KCs) address critical limitations of cellular therapies, including tumorigenic potential and immune rejection [137]. In deep partial-thickness burn models, iPSC-KC exosomes accelerate wound closure approximately by 40% through miR-762-mediated mechanisms. This miRNA targets the promyelocytic leukemia (PML) tumor suppressor, which normally suppresses ITGB1 transcription, a critical regulator of keratinocyte-ECM interactions and directional migration. By inhibiting PML, miR-762 enhances ITGB1 expression, facilitating coordinated collective migration of keratinocytes and endothelial cells [138]. This approach eliminates the risks associated with whole-cell transplantation while maintaining therapeutic efficacy.

**Figure 7:**
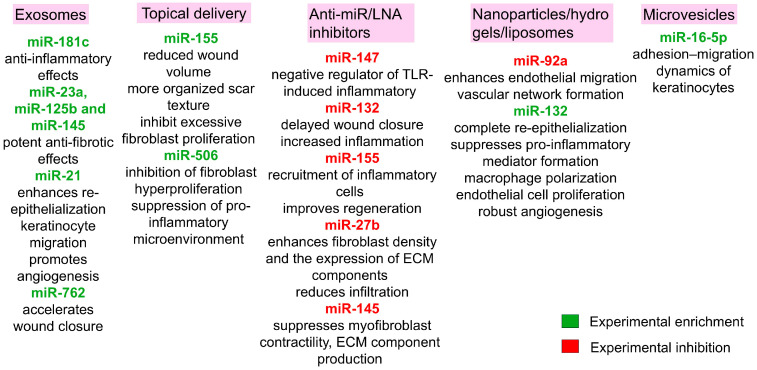
Schematic overview of microRNA-based therapeutic approaches for burn wound healing.

The therapeutic application of microvesicles enriched with miR-16-5p represents a promising strategy for promoting burn wound healing by modulating the adhesion-migration dynamics of keratinocytes. In second-degree burn model, iPSC-derived microvesicles deliver miR-16-5p to wound margins, where it induces a phenotypic shift in epidermal cells through specific molecular targeting. The miR-16-5p direct binds to the 3′-untranslated region of Desmoglein 3 (DSG3) mRNA. DSG3 encodes a desmosomal cadherin essential for intercellular adhesion through intermediate filament anchoring, and its downregulation impairs desmosome assembly, reducing cell–cell adhesion and thereby facilitating collective keratinocyte migration. This adhesion–migration switch is mediated through activation of the p38/MAPK signaling pathway [139]. Importantly, the treatment preserved partial DSG3 expression, which may help to prevent excessive loss of adhesion and pathological acantholysis, while still promoting enhanced migratory capacity. The microvesicles provided additional advantages, including high miR-16-5p loading efficiency and improved retention within burn tissue due to their relatively larger size. Overall, these findings highlight the therapeutic potential of harnessing endogenous regulatory networks, where the inverse relationship between miR-16-5p and DSG3 can be exploited to fine-tune keratinocyte dynamics during wound repair [140]. To summarize the therapeutic platforms considered in this paragraph, Figure 7 schematically depicts delivery vehicles, representative miRNA cargos and expected outcomes.

Burn injury induces a characteristic hyperglycemic response resulting from complex metabolic alterations, including enhanced gluconeogenesis, accelerated glycogenolysis and systemic insulin resistance. This dysregulation stems from excessive release of catecholamines, cytokines and acute-phase proteins, though the precise mechanisms remain incompletely elucidated. Clinically, sustained glucose levels exceeding 11 mM (significantly above the normal 3.3–5.5 mM range) are associated with increased risks of infectious complications such as bacteremia and pneumonia, impaired re-epithelialization and a shift toward catabolic metabolism that collectively contribute to poor outcomes in burn patients [141].

Emerging evidence implicates microRNAs as key regulators of this metabolic dysfunction. miR-194 has been identified as one inducer of hyperglycemia through suppression of IGF1R synthesis [142]. More recently, miR-let-7b has also been recognized as a potent post-transcriptional repressor of IGF1R, with expression levels significantly elevated in burn tissue compared to both miR-194 and non-burned controls. In preclinical models, daily administration of an miR-let-7b inhibitor via tail vein injection for one week prior to burn injury modeling prevented the post-burn decrease in IGF1R, Akt and glycogen synthase kinase-3 beta (Gsk3β) levels observed three days post-injury, demonstrating this miRNA’s role in attenuating PI3K/Akt and Gsk3β activation, critical pathways for maintaining glucose homeostasis [143].

Current therapeutic strategies focus on insulin administration to mitigate hyperglycemia, which improves survival rates and reduces bacterial burden. However, insulin monotherapy provides only short-term glycemic control without addressing underlying IGF1R signaling impairment. A promising alternative approach combines topical insulin with localized delivery of miR-let-7b inhibitors. This dual strategy addresses both immediate glucose management and long-term IGF1R pathway restoration. The enhanced efficacy stems from mechanistic synergy: insulin provides immediate glycemic control while miR-let-7b inhibition prevents the degradation of IGF1R mRNA, enhancing insulin sensitivity and reactivating PI3K/Akt/Gsk3β signaling to promote glucose uptake and glycogen synthesis. This approach not only manages glucose levels but also reprograms metabolism from catabolic to anabolic states and reduces pro-inflammatory cytokine production exacerbated by hyperglycemia [144].

## 9. Current Limitations and Future Directions of microRNA Therapy After Burn

Being important regulators of skin regeneration, microRNAs represent promising candidates for novel therapeutic strategies that address the growing need for safe and effective wound healing treatments [145]. However, miRNA-based therapies face substantial challenges in effective targeting and delivery. Native miRNAs exhibit insufficient stability for in vivo delivery without vectors or chemical modifications due to rapid degradation by endogenous ribonucleases. Their negative surface charge further impedes cellular uptake because of electrostatic repulsion with negatively charged cell membranes. Additional complications include potential off-target effects and the frequent need for repeated administration to achieve sustained therapeutic outcomes. Developing effective delivery systems requires overcoming multiple extracellular and intracellular barriers while avoiding immune activation. To date, various viral and non-viral delivery platforms—including liposomes, cationic polymers, nanoparticles and inorganic materials—have been developed and validated as biovectors for miRNA delivery in diverse pathological conditions [146].

Despite this progress, delivering nucleic acids into the skin, particularly to its deep layers to ensure full regeneration and prevent pathological scarring, remains a complex task. Physical methods such as cavitational ultrasound, electroporation, iontophoresis, subcutaneous injection and microneedles have been employed to enhance penetration [147]. In the context of a burn, despite the disruption of the skin’s barrier layers, targeted delivery is also problematic due to the dense ECM, rapid opsonization of the carrier, rapid uptake by immune cells and a large number of proteases that promote the degradation of protein ligands exposed on the surface of the carrier.

Recent regulatory and scientific advancements highlight promising avenues. The U.S. FDA has approved a transdermal gene delivery system for dystrophic epidermolysis bullosa, targeting partially damaged skin. A notable example is the use of transferosomes to deliver an antimicrobial agent to the deep layers of skin in burn wounds [148]. However, their scalability and stability for nucleic acid delivery remain significant obstacles. Alternative strategies include cell-penetrating peptides, though their potential toxicity and immunogenicity require careful consideration [149]. A particularly promising approach combines a polymeric wound dressing, which protects the wound and maintains a favorable microenvironment, with an encapsulated carrier for therapeutic oligonucleotides [150]. For instance, Li et al. demonstrated that DOTAP/DODAP-based lipid nanoparticles (LNPs) dispersed in a hydrogel could selectively deliver an LNA antagomiR to keratinocytes, significantly accelerating wound closure and restoring barrier function. Notably, their work suggests that a neutral LNP charge may reduce immune cell uptake, challenging the prevailing belief that a cationic charge is optimal for transdermal delivery [151].

Nonetheless, despite significant advances in bioengineering techniques, the path to clinical implementation of microRNA-based treatments requires rigorous characterization of multiple parameters before specific miRNAs can be deployed as targeted therapeutics. This process begins with comprehensive expression profiling to identify pathology-associated miRNA signatures, followed by in vitro efficacy screening of candidate constructs and extensive in vivo pharmacokinetic and safety studies prior to potential clinical translation. Currently, several miRNA-based therapeutics have advanced through preclinical development, with a subset meeting the requirements for clinical trials in wound healing applications. Notably, some candidates have already entered human trials. Leading this translational effort, MiRagen Therapeutics has advanced two promising candidates: Remlarsen (MRG-201), a synthetic miR-29b mimic with completed Phase II trials (NCT03601052) for reducing fibrotic scarring through coordinated suppression of collagen types I, III and IV, as well as other extracellular matrix proteins involved in pathological scarring [152], and MRG-110, an inhibitor of miR-92a in the completed Phase I (NCT03603431) that promotes angiogenesis by enhancing endothelial cell function and stimulating new blood vessel growth to improve wound perfusion and healing [153].

However, significant challenges remain, particularly concerning biological redundancy and compensatory mechanisms within miRNA networks. The inherent redundancy often limits the efficacy of single-miRNA targeting, as other miRNAs may functionally compensate for the inhibited or supplemented molecule. This compensatory capacity can attenuate the desired therapeutic effect on protein output and downstream signaling cascades. Consequently, there is a growing recognition that effective miRNA therapeutics may require multi-target strategies employing carefully designed miRNA panels to simultaneously modulate several components of a pathological network [154].

This need for a comprehensive approach is critically underscored by the very nature of pathological remodeling in severe wounds. Such remodeling is rooted in a fundamental disruption of healing dynamics, which is characterized by a prolonged inflammatory phase, an excessive proliferative phase that drives aberrant extracellular matrix synthesis and an ultimately impaired remodeling phase. Therefore, a successful therapeutic strategy must extend beyond targeting a single molecular process. Instead, it necessitates a holistic intervention that can orchestrate the normal progression of all regenerative phases, particularly in the context of deep and extensive burns.

## 10. Conclusions

Burn injuries remain a significant global health burden, with millions of severe cases reported annually involving damage to underlying tissues and organs. Despite considerable advances in critical care and surgical techniques, mortality rates from burn-related complications remain unacceptably high, underscoring the urgent need for novel therapeutic approaches that can improve regeneration and mitigate systemic responses in burn patients [155].

MicroRNAs have emerged as molecular regulators with transformative potential in regenerative medicine due to their roles in cellular behavior under both physiological and pathological conditions. In the context of burn injury, specific miRNAs demonstrate therapeutic promise through their ability to modulate key pathological processes: they fine-tune inflammatory responses to prevent excessive tissue damage while maintaining essential immune functions, promote angiogenesis to ensure adequate oxygen and nutrient delivery to healing tissues, regulate keratinocyte migration and proliferation during epithelial restoration phases and coordinate extracellular matrix deposition and remodeling to minimize pathological scarring [156]. The capacity of miRNAs to simultaneously regulate multiple genes within functional networks provides a distinct advantage over single-target therapies, enabling coordinated intervention in complex pathological cascades. This multi-target approach is particularly valuable in burn care, where successful outcomes require synchronized modulation of inflammation, angiogenesis, epithelialization and tissue remodeling.

The therapeutic promise of microRNAs is increasingly being realized through synergistic advancements in two areas: the development of delivery systems that overcome biological barriers and the innovative integration of active biomolecules into biocompatible scaffolds and hydrogels that enable sustained, localized release at wound sites. These parallel developments have accelerated clinical translation, with multiple miRNA-based candidates now advancing through Phase I/II trials with encouraging preliminary results [157]. Looking forward, several strategic directions can promise to further enhance the efficacy and applicability of miRNA therapeutics in burn care [158].

However, the development of these therapies must be guided by a nuanced understanding of biological complexity. A critical consideration is that a single microRNA can exert opposing effects on the diverse cell populations present in the skin. Furthermore, the systemic impact of a severe burn can significantly influence the local wound microenvironment and, consequently, the efficacy of a targeted therapy. Therefore, a successful treatment strategy must account for both this cellular context-dependence and the systemic pathophysiology of the injury.

## Figures and Tables

**Table 1:** Comparison of burn wound therapies: application, advantages, disadvantages and limitations.

Therapy	Typical Application	Advantages	Disadvantages	Limitations (Practical Notes)	References
Cooling with running water	Acute care of superficial and partial-thickness burns within first minutes	Reduces heat penetration, pain and depth progression; widely available and low cost	Limited to very early window (first 10–20 min) for maximal effect; insufficient for deep burns	Universal recommendation for initial management; avoid ice or prolonged refrigeration, which can worsen injury	[21]
Simple dressings and emollients	Superficial burns; temporary coverage	Inexpensive, easy to apply; maintain basic moist environment	Require frequent changes; limited antimicrobial activity; can adhere if improper	Suitable for small superficial burns; not optimal for exudative or infected wounds	[41]
Semipermeable film dressings (polyurethane: Tegaderm)	Superficial and some partial-thickness burns	Maintain moist microenvironment; permit wound inspection without removal; reduce pain	Not appropriate for heavily exuding wounds; potential for maceration if misused	Useful for small superficial burns and donor sites; contraindicated with high exudate or established infection	[42]
Hydrogels (amorphous gels and sheet hydrogel: IntraSite)	Partial-thickness burns and necrotic or dry wounds	Rehydrate necrotic tissue; facilitate autolytic debridement	Require secondary absorbent dressing for exudative wounds; may need frequent reapplication	Good for maintaining moist environment and pain control	
Hydrocolloid dressings (DuoDERM)	Low-to-moderate exudate partial-thickness wounds	Promote autolytic debridement; long wear time	Can be difficult to remove; not ideal for infected wounds	Effective where exudate is controlled and infection risk low	
Foam dressings (Mepilex)	Moderate-to-high exudate partial-thickness wounds	High absorbency; cushioning; reduced dressing change pain	Less transparent (harder to inspect wound)	Often used under secondary dressings or NPWT pads	
Antimicrobial dressings: silver (AQUACEL)	Partial-thickness burns at risk of infection	Broad-spectrum antimicrobial effect; prolonged activity; can reduce infection rates	Potential cytotoxicity at high concentrations	Indicated for contaminated wounds or high infection risk	[43]
Enzymatic debridement	Early selective debridement of deep partial-thickness and some full-thickness eschar	Selective removal of necrotic tissue; potentially reduces need for surgery; may preserve viable tissue	Pain during application in some patients; strict application protocols	Regional regulatory status varies; appropriate for controlled settings with experienced staff	[44]
Early tangential/surgical excision and autografting	Deep partial-thickness to full-thickness burns	Rapid removal of necrotic tissue, decreases infection risk, shortens hospital stay and definitive closure with autologous skin	Requires operating room; donor-site morbidity; risk of graft failure/infection	Gold standard for full-thickness burns and large, deep wounds; timing and technique tailored to patient stability	[12]
Negative-pressure wound therapy	Adjunct for graft fixation, large open wounds and exudative wounds	Promotes granulation tissue, reduces edema and bacterial load and secures grafts improving take	Device cost; requires vacuum system and trained personnel	Widely used as adjunct to surgical and dressing management; portable systems facilitate mobility	[37]
Platelet-rich plasma	Adjunct therapy for deep and partial-thickness burns	Delivers endogenous growth factors (PDGF, TGF-β, VEGF, FGF and EGF); accelerates epithelialization	Variability in preparation; limited standardization	Widely used autologous biotherapy; approved in multiple jurisdictions	[45]
Recombinant growth factor therapy	Chronic or delayed-healing burn wounds	Stimulates fibroblast proliferation, angiogenesis and epithelialization	Limited depth penetration; potential mitogenic risk; too-rapid degradation and clearance of GFs	FDA and EMA approved for chronic wounds; used off-label for burns	[46]
Bioengineered skin substitutes (Integra and Apligraf)	Deep partial- and full-thickness burns requiring dermal replacement	Provide dermal matrix scaffold, reduce graft requirement and promote neovascularization	Expensive and require careful storage	Clinically approved acellular and composite grafts	[47]
Cultured epithelial autografts (CEAs)	Extensive burns with limited donor sites	Permanent coverage using patient’s cells; reduces donor-site trauma	High cost; time for culture preparation; poor graft takes and stability	FDA approved (Epicel); used for extensive burns >30% TBSA	[38]
Allogeneic cell-based dressings	Temporary coverage in extensive burns	Provide bioactive molecules promoting healing	Immunological risk	Bridging therapy prior to autografting; facilitate tissue salvage by itself	[48]
Gene-activated matrix dressings	Advanced molecular therapy for severe burns	Sustained local release of angiogenic and reparative signals	High cost; specialized use; early regulatory adoption	Approved in selected markets (e.g., China) as advanced wound care devices	[49]

FDA—Food and Drug Administration; EMA—European Medicines Agency.

**Table 2:** MicroRNA representation in burn injury and during its regeneration phases.

Phase	microRNA	Effect on Burn Wound Healing	Reported Manipulation	References	Other Pathological Conditions
Hemostasis	miR-143/145	Necessary for differentiation of vascular smooth muscle cells, as well as for functional vasoconstriction and vasodilation	Knockout mice	[56]	Atherosclerosis; epithelial cancers; B-cell malignancies; coronary artery disease (all ↓)
	miR-15b	Promotes proliferation of vascular smooth muscle cells	5 nM miRNA mimic (chemically modified double-stranded RNA) 40 nM anti-miR (2′-*O*-methyl-modified RNA oligonucleotides)	[57]	Cardiac ischemia injury (↑); pulmonary fibrosis (↑); tongue squamous cell carcinoma
	miR-221	Promotes proliferation and differentiation of vascular smooth muscle cells by inhibiting *c-kit* expression	0.3 or 3 nm (chemically modified double-stranded RNA) 106 nm anti-miR (2′-*O*-methyl-modified RNA oligonucleotides)	[58]	Highly expressed in cancer-derived cells; inhibits normal erythropoiesis
	miR-409-3p/29	Suppresses fibrinogen synthesis	30 nM miRNA precursor molecules	[59]	miR-409-3p: cardiac fibrosis and acute coronary syndrome (↑); type 1 diabetes (↓) miR-29: pulmonary fibrosis (↓); osteosarcoma (↑)
	miR-98	Enhances endotheliocyte permeability by suppressing the synthesis of hypoxia-inducible factor 1-alpha (HIF-1α) inhibitor protein	10 to 50 nM anti-miR	[60]	Systemic lupus erythematosus; osteoarthritis; stroke (all ↓)
Inflammation	miR-424	Differentiation of monocytes into macrophages; activation of the macrophage colony-stimulating factor receptor (M-CSFR) gene due to suppression of nuclear factor I type A (NFI-A) translation	-	[61]	Hepatocellular carcinoma (↓); deep vein thrombosis (↑); obesity (↑)
	miR-21	Enhances efferocytosis; suppresses the activation of NF-kB and synthesis of TNF-α; enhances the production of IL-10	-	[62]	Contributes to cardiac fibrosis; acute myocardial infarction; stroke; atherosclerosis (all ↑)
	miR-147	Anti-inflammatory effect; regulates toll-like receptor (TLR)-induced inflammatory reactions in macrophages	40 nM miRNA mimic and 40 nM locked nucleic acid (LNA) miR-147 inhibitor	[63]	Rheumatoid arthritis (↑); pulmonary tuberculosis (↑); coronary artery disease (↓)
	miR-155	Pro-inflammatory effect; enhances the production of TNF-α/IL-6 and the differentiation of macrophages into the M1	Knockout mice	[64]	Multiple sclerosis, Alzheimer’s disease; atherosclerosis; heart failure (all ↑)
	miR-183-3p	Regulation of skin capillary permeability and inflammatory mediators release	In vivo burned skin model; qRT-PCR analysis	[65]	Non-small-cell lung carcinoma (↑); psoriasis (↓)
	miR-132	Reduces the production of chemokines by keratinocytes; limits the excessive production of pro-inflammatory cytokines by macrophages; polarization of macrophages into M2	20 nM pre-miR-132 and 20 nM LNA inhibitor	[66,67]	Alzheimer’s disease; heart failure; post-traumatic stress disorder (all ↑)
	miR-27b	Inhibits fibroblast proliferation	4 µg/kg mimic; 4 µg/kg inhibitor	[68]	Kawasaki disease (↑); breast and prostate cancers (↓)
Proliferation	miR-126	Induces neoangiogenesis	-	[69]	Atherosclerosis (↓); acute myocardial infarction (↓); aerobic exercises (↑); knee osteoarthritis (↑)
	miR-21	Promotes migration of keratinocytes and fibroblasts	miR-21 antagomir (16 μg dissolved in 100 μL of PBS); 20 μg of miR-21 plasmid DNA	[70]	Contributes to cardiac fibrosis; acute myocardial infarction; stroke; atherosclerosis (all ↑)
	miR-31	Promotes keratinocytes proliferation and migration	20 nM miR-31 precursor	[71]	Diabetic wounds (↓); oral cancer (↑)
	miR-198	Suppresses keratinocytes proliferation and migration	-	[72]	Pancreatic cancer (↓); osteosarcoma (↓); non-healing diabetic ulcers (↑)
	miR-99 family		20 μM mimic; 20 μM LNA-inhibitor	[73]	Cardiac hypertrophy (↑); enhances HBV replication; highly expressed in hematopoietic and acute myeloid leukemia stem cells
	miR-210	Suppresses keratinocyte proliferation; promotes angiogenesis	500 nM LNA-based anti-miR	[74]	Cardiac stress (↑); solid tumors (↑); retinal degeneration (↓)
	miR-199a-5p	Suppresses angiogenesis	Mimic (50 nm); inhibitor (100 nm)	[75]	Fibrosis (↑); myocardial infarction (↓)
	miR-130a	Inhibits re-epithelialization and granulation tissue formation; promotes angiogenesis	5 μM mimic	[76]	Endothelial cell senescence; diabetic vascular disease; ischemic stroke (all ↓)
	miR-203	Inhibits keratinocytes proliferation and migration	80 mg/kg	[77]	Melanoma (↓); psoriasis (↑); ER-positive breast cancer (↑)
	miR-200b	Activation of angiogenic transcription factor ETS-1 and intensification of angiogenesis	50 nM mimic; 100 nM inhibitor	[78]	Lung cancer; nonalcoholic fatty liver disease (all ↓)
	miR-499-5p	Impairs the angiogenic properties of endothelial cells and reduces blood perfusion	3 μL/mL mimic 3 μL/mL inhibitor	[79]	Myocardial infarction (in ischemic tissue ↓; in the blood ↑)
	miR-663	Inhibit apoptosis and promote proliferation of fibroblasts and keratinocytes	-	[80]	Hematologic malignancies (↓); non-small-cell lung carcinoma (↑); vascular smooth muscle cells differentiation (↑)
	miR-486				Non-small-cell lung carcinoma; Duchenne muscular dystrophy (all ↓)
	miR-23b	Promotes fibroblast proliferation and migration through Smad3	-	[81]	Rheumatoid arthritis; ovarian cancer; Parkinson’s disease (all ↓)
	miR-27b	Suppresses the directed migration of mesenchymal stem cells from bone marrow to damaged tissues by regulating the synthesis of stromal cell-derived factor-1 (SDF-1a)	100 nM miRNA mimic	[82]	Kawasaki disease (↑); breast and prostate cancers (↓)
	miR-let-7c	Inhibits the proliferation and migration of dermal fibroblasts by binding to heat shock protein 70 (HSP70), increasing Bcl-2 and decreasing Bax level	50 nM mimic and inhibitor	[83]	Prostate cancer (↓); coronary artery disease (↓); predictor of acute chest syndrome (↑)
Remodeling	miR-1908	Increases production of TGF-β1, IL-1a, TNF-α and collagen I by fibroblasts	2 μg/kg mimic and inhibitor	[84]	Glioblastoma (↑); myocardial infarction (↓); HBV infection (↑)
	miR-29b-3p	Affects the expression of collagen genes to improve extracellular matrix remodeling	-	[85]	Pre-eclampsia (↑); congenital heart disease (↑); prostate cancer (↓)
	miR-29a	Reduces the levels of collagen type I	100 nM mimic and 2′-OMe chemically modified inhibitors	[86]	Metastatic prostate cancer; oral squamous carcinoma; cardiac fibrosis (all ↓)
	miR-145	Decreases Krüppel-like factor 4 (KLF4) level, thereby suppressing α-SMA synthesis and fibroblast differentiation into myofibroblasts	5 nM inhibitor	[87]	Prostate cancer; coronary artery disease; type-2 diabetes mellitus (all ↓)
	miR-let-7d	Reduction of iron absorption through divalent metal transporter 1 (DMT1) and stabilization of new collagen production	100 nM mimic and 100 nM anti-miR	[88]	Fibromyalgia syndrome (↑); idiopathic pulmonary fibrosis (↓)
	miR-506-3p	Regulates the autophagy of fibroblasts, their migration and proliferation and the synthesis of ECM components	-	[89]	Osteosarcoma (↓); hepatic steatosis (↑)

↑—overexpression of microRNA; ↓—decreased expression of microRNA.

## Data Availability

No new data were created or analyzed in this study. Data sharing is not applicable to this article.
